# Crystal structure of 2-isopropyl-5,7′-dimethyl-1′,3′,3a’,6′,8a’,8b’-hexa­hydro­spiro­[cyclo­hexane-1,6′-furo[3,4-*d*]imidazo[1,5-*b*]isoxazol]-8′(7′*H*)-one

**DOI:** 10.1107/S2056989016010641

**Published:** 2016-07-19

**Authors:** Heithem Abda, Khaireddine Ezzayani, Kaiss Aouadi, Taha Guerfel, Sebastien Vidal, Moncef Msaddek

**Affiliations:** a University of Monastir, Heterocyclic Chemistry Laboratory, Products, Natural and Reactivity, Faculty of Sciences of Monastir, Avenue of the Environment, 5000 Monastir, Tunisia; b University of Monastir, Laboratory of Physical Chemistry of Materials, Faculty of Sciences of Monastir, Avenue of the Environment, 5019 Monastir, Tunisia; c Laboratory of Electrochemistry, Materials and Environment, Kairouan University, 3100 Kairouan, Tunisia; d University of Lyon CNRS, Institute of Chemistry and Biochemistry and Molecular Supramolecular, UMR 5246, Laboratoire de Chimie Organique 2-Glycochemistry, Curien Building, 43 Boulevard du 11 Novembre 1918, F-69622 Villeurbanne, France

**Keywords:** crystal structure, isoxazolidine, hydrogen bonding

## Abstract

In the title compound, C_17_H_28_N_2_O_3_, the isoxazolidine ring adopts an envelope conformation with the O atom deviating from the mean plane of the other four ring atoms by 0.617 (1) Å. In the crystal, mol­ecules are linked *via* weak C—H⋯O hydrogen bonds, forming chains which extend along the *b*-axis direction.

## Abstract 2

Cyclo­addition reaction between a menthone-based chiral nitrone and 2,5-di­hydro­furan under microwave activation affords a enanti­opure cyclo­adduct in good yields and with high stereoselectivity

## Chemical context   

The 1,3-dipolar cyclo­addition of nitro­nes to alkenes has been applied to produce substituted isoxazolidines (Gothelf & Jørgensen, 1998 ▸). These compounds can be converted into *β*-amino alcohols (Padwa *et al.*, 2002 ▸), *β*-lactams (Hanselmann *et al.*, 2003 ▸) and *α*-amino acids (Aouadi *et al.*, 2006 ▸), by reductive cleavage of the N—O bond. Consequently, isoxazol­idines have been used as key inter­mediates for the synthesis of various natural products or anti­fungal, anti-inflammatory, anti-mycobacterial, anti-tuberculosis and anti­viral agents. The previously mentioned importance of the isoxazolidine substructure led us to investigate the cyclo­addition of chiral nitrone [(5(*S*),6(*S*),9(*R*)-6-isopropyl-4,9-dimethyl-3-oxo-1,4-di­aza­spiro­[4.5]dec-1-ene-1-oxide] with 2,5-di­hydro­furan. The present work reports the synthesis and the X-ray crystallographic study of this substituted isoxazol­idine, the title compound, C_17_H_28_N_2_O_3_, (I).
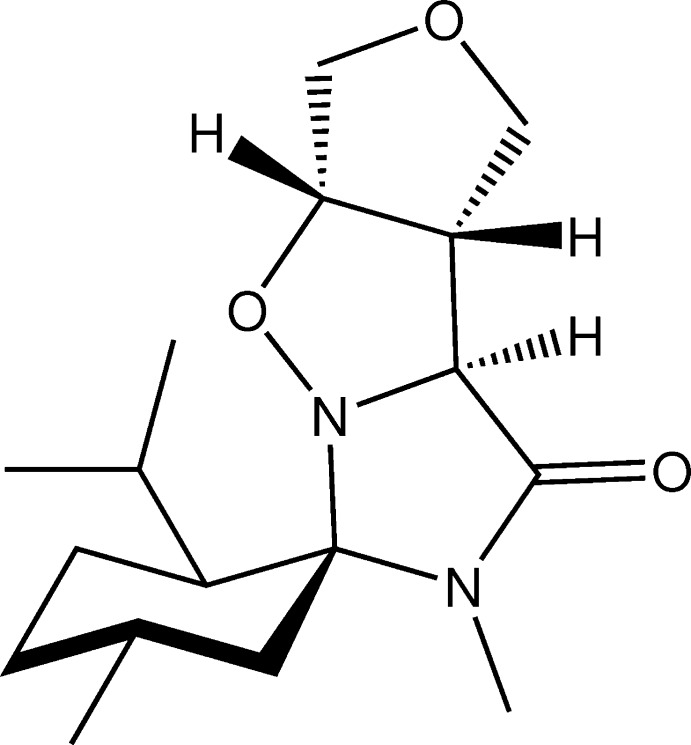


## Structural commentary   

**Figure 1:**
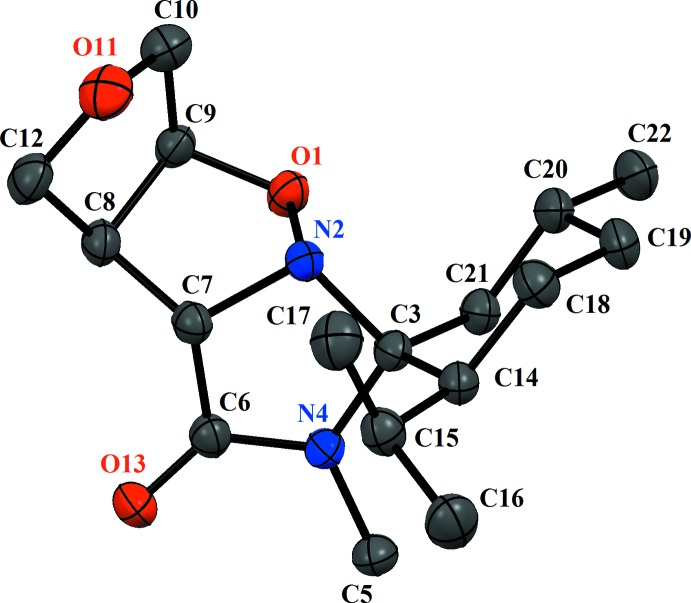
The mol­ecular conformation in the mol­ecules of (I), showing the atom labelling. Displacement ellipsoids are drawn at the 35% probability level. H atoms have been omitted for clarity.

In the title compound (I), the asymmetric unit comprises a single mol­ecule (Fig. 1 ▸). Each mol­ecule has six stereogenic centres (Abda *et al.*, 2014 ▸) although the absolute configuration for the mol­ecule was not determined definitively in this analysis. The isoxazolidine ring (O1/N2/C7–C9) adopts an envelope conformation with atom O1 displaced by 0.617 (1) Å from the mean plane through atoms N2/C7–C9. The N—O bond lengths of the isoxazolidine rings O1—N2 = 1.482 (2) Å, close to values reported for related compounds (Loh *et al.*, 2010 ▸; Molander *et al.*, 2013 ▸).

## Supra­molecular features   

**Figure 2:**
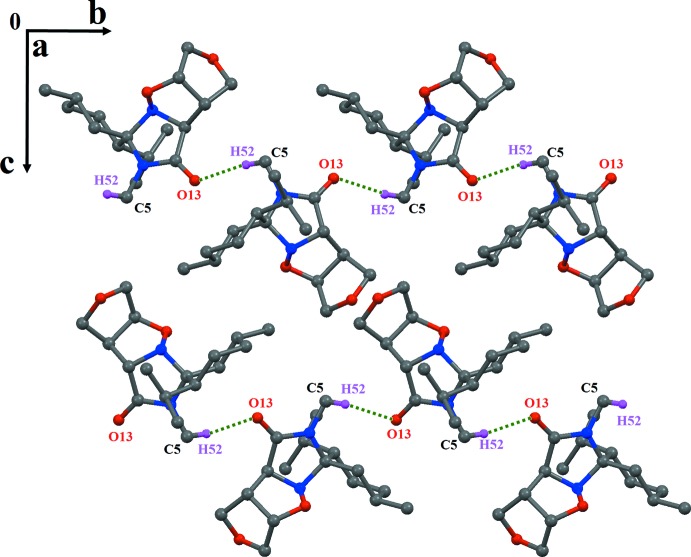
The C—H⋯O hydrogen-bonded chains extending along the *b* axis in the crystal structure of (I). Dashed lines indicate hydrogen bonds. Non-associated H atoms have been omitted.

In the crystal, the mol­ecules are linked *via* non-classical weak C5—H52⋯O13^i^ hydrogen bonds, forming zigzag chains, which extend along the *b-*axis direction (Table 1 ▸ and Fig. 2 ▸).

## Synthesis and crystallization   

**Figure 3:**
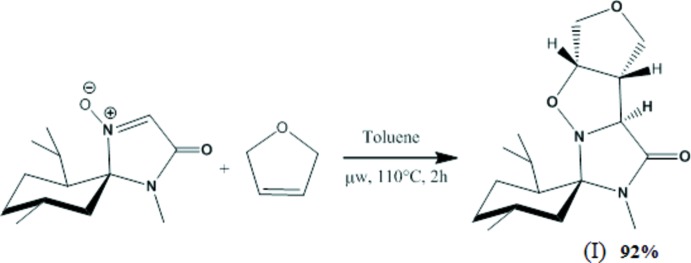
The cyclo­addition reaction in the synthesis of (I).

In a Biotage Initiator 10 ml vial, nitrone [(5(*S*),6(*S*),9(*R*)-6-isopropyl-4,9-dimethyl-3-oxo-1,4-di­aza­spiro­[4.5]dec-1-ene-1-oxide] (1 eq.) in anhydrous toluene (4 ml) was introduced. The vial was flushed with argon and 2,5-di­hydro­furan (3 eq,) was added. The vial was sealed with a septum cap and was irradiated with microwaves (temperature: 373 K) (Fig. 3 ▸). TLC monitoring (EtOAc/PE 5/5) showed full conversion after 2 h. After the crude mixture was concentrated and purified by flash column chromatography (silica gel, EtOAc/PE 5/5), the desired isoxazolidine (I) was obtained (m.p. = 410–411 K).

## Refinement   

Crystal data, data collection and structure refinement details are summarized in Table 2 ▸. The H atoms were located in a difference map, but these were repositioned geometrically and were initially refined with soft restraints on the bond lengths and angles to regularize their geometry (C—H in the range 0.93–0.98 Å) and *U*_iso_(H) (in the range 1.2–1.5 times *U*_eq_ of the parent atom). These were subsequently refined with riding constraints (Cooper *et al.*, 2010 ▸). Although not definitive for this chiral structure, the Flack (1983 ▸) absolute structure parameter obtained [0.60 (3) for 1261 Friedel pairs] gave C3(*S*), C7(*S*), C8(*S*), C9(*S*), C14(*S*), C20(*R*) assignments for the six arbitrarily named chiral centres in the mol­ecule. The inverted structure gave a similarly high Flack factor .

## Supplementary Material

Crystal structure: contains datablock(s) global, I. DOI: 10.1107/S2056989016010641/zs2363sup1.cif

Structure factors: contains datablock(s) I. DOI: 10.1107/S2056989016010641/zs2363Isup2.hkl

Supporting information file. DOI: 10.1107/S2056989016010641/zs2363Isup3.cml

CCDC reference: 1489217

Additional supporting information: crystallographic information; 3D view; checkCIF report

## Figures and Tables

**Table 1:** Hydrogen-bond geometry (Å, °)

*D*—H⋯*A*	*D*—H	H⋯*A*	*D*⋯*A*	*D*—H⋯*A*
C5—H52⋯O13^i^	0.97	2.57	3.536 (3)	172

Symmetry code: (i) 
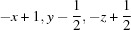
.

**Table 2:** Experimental details

Crystal data	
Chemical formula	C_17_H_28_N_2_O_3_
*M*_r_	308.42
Crystal system, space group	Orthorhombic, *P*2_1_2_1_2_1_
Temperature (K)	150
*a*, *b*, *c* (Å)	7.7474 (6), 11.1404 (8), 19.208 (2)
*V* (Å^3^)	1657.8 (2)
*Z*	4
Radiation type	Cu *K*α
μ (mm^−1^)	0.68
Crystal size (mm)	0.49 × 0.43 × 0.25

Data collection	
Diffractometer	Oxford Diffraction Xcalibur (Atlas, Gemini Ultra)
Absorption correction	Analytical [*CrysAlis PRO* (Agilent, 2013 ▸) based on expressions derived by Clark & Reid (1995 ▸); changes in illuminated volume were kept to a minimum, and were taken into account (Görbitz, 1999 ▸)]
*T*_min_, *T*_max_	0.782, 0.866
No. of measured, independent and observed [*I* > 2.0σ(*I*)] reflections	10374, 2879, 2680
*R*_int_	0.059
(sin θ/λ)_max_ (Å^−1^)	0.596

Refinement	
*R*[*F* ^2^ > 2σ(*F* ^2^)], *wR*(*F* ^2^), *S*	0.042, 0.096, 1.03
No. of reflections	2866
No. of parameters	201
H-atom treatment	H-atom parameters constrained
Δρ_max_, Δρ_min_ (e Å^−3^)	0.16, −0.17
Absolute structure	Flack (1983 ▸), 1261 Friedel pairs
Absolute structure parameter	0.6 (3)

Computer programs: *CrysAlis PRO* (Agilent, 2013 ▸), *SIR97* (Altomare *et al.*, 1999 ▸), *CRYSTALS* (Betteridge *et al.*, 2003 ▸), *CAMERON* (Watkin *et al.*, 1996 ▸), Larson (1970 ▸), Prince (1982 ▸) and Watkin (1994 ▸).

## supplementary crystallographic information

### Crystal data

**Table:**

C_17_H_28_N_2_O_3_	*F*(000) = 672
*M**_r_* = 308.42	*D*_x_ = 1.236 Mg m^−^^3^
Orthorhombic, *P*2_1_2_1_2_1_	Cu *K*α radiation, λ = 1.5418 Å
Hall symbol: P 2ac 2ab	Cell parameters from 5548 reflections
*a* = 7.7474 (6) Å	θ = 4.5–66.7°
*b* = 11.1404 (8) Å	µ = 0.68 mm^−^^1^
*c* = 19.208 (2) Å	*T* = 150 K
*V* = 1657.8 (2) Å^3^	Block, colorless
*Z* = 4	0.49 × 0.43 × 0.25 mm

### Data collection

**Table:**

Oxford Diffraction Xcalibur (Atlas, Gemini Ultra) diffractometer	2879 independent reflections
Radiation source: Enhance Ultra (Cu) X-ray source	2680 reflections with *I* > 2.0σ(*I*)
Mirror monochromator	*R*_int_ = 0.059
Detector resolution: 10.4678 pixels mm^-1^	θ_max_ = 66.8°, θ_min_ = 11°
ω scans	*h* = −9→8
Absorption correction: analytical [CrysAlis PRO (Agilent, 2013) based on expressions derived by Clark & Reid (1995); changes in illuminated volume were kept to a minimum, and were taken into account (Görbitz, 1999)]	*k* = −13→12
*T*_min_ = 0.782, *T*_max_ = 0.866	*l* = −22→21
10374 measured reflections	

### Refinement

**Table:**

Refinement on *F*^2^	H-atom parameters constrained
Least-squares matrix: full	Method, part 1, Chebychev polynomial, (Watkin, 1994: *P*rince, 1982) [*w*eight] = 1.0/[A_0_*T_0_(x) + A_1_*T_1_(x) ··· + A_n-1_]*T_n-1_(x)] where A_i_ are the Chebychev coefficients listed belo*w* and x = *F* /*F*max Method = Robust Weighting (*P*rince, 1982) W = [*w*eight] * [1-(delta*F*/6*sigma*F*)^2^]^2^ A_i_ are: 0.124E + 04 0.195E + 04 0.105E + 04 304.
*R*[*F*^2^ > 2σ(*F*^2^)] = 0.042	(Δ/σ)_max_ = 0.0002
*wR*(*F*^2^) = 0.096	Δρ_max_ = 0.16 e Å^−^^3^
*S* = 1.03	Δρ_min_ = −0.17 e Å^−^^3^
2866 reflections	Extinction correction: Larson (1970), Equation 22
201 parameters	Extinction coefficient: 74 (4)
0 restraints	Absolute structure: Flack (1983), 1261 Friedel pairs
Primary atom site location: structure-invariant direct methods	Absolute structure parameter: 0.6 (3)
Hydrogen site location: difference Fourier map	

### Special details

**Table:**

Experimental. The crystal was placed in the cold stream of an Oxford Cryosystems open-flow nitrogen cryostat with a nominal stability of 0.1K.
Refinement. The analytical numeric absorption correction using a multi-faceted crystal model is based on expressions derived by Clark & Reid (1995). The relatively large ratio of minimum to maximum corrections applied in the multi-scan process (1:nnn) reflect changes in the illuminated volume of the crystal. Changes in illuminated volume were kept to a minimum, and were taken into account (Görbitz, 1999).

### Fractional atomic coordinates and isotropic or equivalent isotropic displacement parameters (Å^2^)

**Table:**

	*x*	*y*	*z*	*U*_iso_*/*U*_eq_	
O1	0.3924 (2)	0.46528 (13)	0.43226 (8)	0.0295	
N2	0.2276 (2)	0.48439 (15)	0.39468 (9)	0.0248	
C3	0.2216 (3)	0.39172 (18)	0.33856 (11)	0.0253	
N4	0.3084 (3)	0.44977 (16)	0.27929 (9)	0.0257	
C5	0.3388 (3)	0.3911 (2)	0.21315 (11)	0.0309	
H51	0.3934	0.4467	0.1812	0.0471*	
H53	0.2321	0.3682	0.1919	0.0466*	
H52	0.4129	0.3213	0.2193	0.0470*	
C6	0.3369 (3)	0.56800 (19)	0.28913 (11)	0.0269	
C7	0.2626 (3)	0.60075 (18)	0.35923 (11)	0.0257	
C8	0.3798 (3)	0.67280 (19)	0.40726 (12)	0.0279	
C9	0.4274 (3)	0.5792 (2)	0.46276 (11)	0.0271	
C10	0.3137 (4)	0.6110 (2)	0.52465 (12)	0.0372	
O11	0.1969 (2)	0.70248 (16)	0.50158 (9)	0.0397	
C12	0.2873 (3)	0.7679 (2)	0.44941 (13)	0.0352	
H122	0.2094	0.8163	0.4202	0.0417*	
H121	0.3689	0.8236	0.4714	0.0416*	
H102	0.2495	0.5396	0.5410	0.0447*	
H101	0.3821	0.6433	0.5635	0.0448*	
H91	0.5499	0.5839	0.4753	0.0337*	
H81	0.4758	0.7027	0.3812	0.0341*	
H71	0.1551	0.6415	0.3518	0.0307*	
O13	0.4052 (2)	0.63710 (15)	0.24780 (8)	0.0357	
C14	0.0307 (3)	0.3616 (2)	0.32135 (11)	0.0274	
C15	−0.0806 (3)	0.4686 (2)	0.29604 (12)	0.0310	
C16	−0.2129 (4)	0.4250 (3)	0.24307 (14)	0.0435	
H162	−0.2845	0.4937	0.2288	0.0654*	
H163	−0.1594	0.3903	0.2017	0.0653*	
H161	−0.2850	0.3629	0.2652	0.0658*	
C17	−0.1731 (4)	0.5369 (2)	0.35403 (14)	0.0406	
H171	−0.2186	0.6122	0.3336	0.0606*	
H173	−0.2672	0.4908	0.3721	0.0604*	
H172	−0.0979	0.5542	0.3931	0.0602*	
H151	−0.0031	0.5263	0.2711	0.0372*	
C18	−0.0536 (3)	0.2924 (2)	0.38122 (13)	0.0346	
C19	0.0460 (4)	0.1795 (2)	0.40142 (14)	0.0379	
C20	0.2339 (3)	0.2100 (2)	0.41916 (12)	0.0327	
C21	0.3154 (3)	0.27553 (19)	0.35817 (12)	0.0288	
H211	0.3096	0.2212	0.3171	0.0339*	
H212	0.4342	0.2949	0.3684	0.0350*	
C22	0.3387 (4)	0.0976 (2)	0.43654 (13)	0.0429	
H222	0.4583	0.1173	0.4469	0.0645*	
H221	0.3358	0.0416	0.3963	0.0629*	
H223	0.2912	0.0549	0.4787	0.0629*	
H201	0.2358	0.2644	0.4599	0.0385*	
H192	0.0469	0.1237	0.3607	0.0463*	
H191	−0.0113	0.1390	0.4418	0.0451*	
H182	−0.1724	0.2721	0.3658	0.0423*	
H181	−0.0606	0.3469	0.4217	0.0410*	
H141	0.0390	0.3055	0.2810	0.0325*	

### Atomic displacement parameters (Å^2^)

**Table:**

	*U*^11^	*U*^22^	*U*^33^	*U*^12^	*U*^13^	*U*^23^
O1	0.0344 (8)	0.0232 (7)	0.0308 (8)	0.0026 (7)	−0.0085 (7)	−0.0014 (6)
N2	0.0304 (10)	0.0202 (9)	0.0239 (9)	0.0002 (8)	−0.0028 (8)	0.0005 (7)
C3	0.0324 (12)	0.0197 (10)	0.0237 (10)	0.0008 (9)	0.0029 (9)	−0.0011 (9)
N4	0.0317 (9)	0.0218 (9)	0.0236 (9)	−0.0001 (8)	0.0035 (8)	−0.0015 (7)
C5	0.0377 (12)	0.0307 (11)	0.0242 (11)	−0.0039 (10)	0.0039 (9)	−0.0034 (9)
C6	0.0290 (11)	0.0233 (10)	0.0284 (11)	−0.0006 (9)	−0.0018 (9)	0.0016 (9)
C7	0.0302 (11)	0.0206 (11)	0.0262 (10)	0.0023 (10)	0.0024 (9)	0.0014 (8)
C8	0.0324 (12)	0.0204 (10)	0.0307 (12)	−0.0016 (9)	0.0028 (10)	−0.0014 (9)
C9	0.0313 (11)	0.0233 (10)	0.0267 (10)	0.0000 (9)	−0.0042 (9)	−0.0040 (9)
C10	0.0453 (14)	0.0361 (13)	0.0302 (12)	−0.0014 (12)	−0.0002 (11)	−0.0035 (10)
O11	0.0392 (10)	0.0387 (9)	0.0413 (9)	0.0061 (8)	0.0076 (8)	−0.0078 (8)
C12	0.0421 (15)	0.0257 (11)	0.0377 (13)	0.0022 (11)	−0.0016 (11)	−0.0076 (10)
O13	0.0489 (10)	0.0280 (8)	0.0303 (8)	−0.0062 (8)	0.0057 (8)	0.0044 (6)
C14	0.0329 (11)	0.0228 (11)	0.0265 (11)	−0.0031 (9)	0.0021 (9)	−0.0005 (9)
C15	0.0295 (11)	0.0298 (12)	0.0335 (12)	−0.0022 (10)	−0.0007 (10)	0.0024 (9)
C16	0.0430 (14)	0.0423 (14)	0.0453 (14)	−0.0056 (13)	−0.0109 (13)	0.0027 (12)
C17	0.0370 (13)	0.0368 (13)	0.0481 (15)	0.0038 (12)	0.0041 (12)	−0.0022 (11)
C18	0.0371 (13)	0.0322 (13)	0.0344 (12)	−0.0047 (11)	0.0039 (11)	0.0025 (10)
C19	0.0533 (16)	0.0238 (12)	0.0365 (13)	−0.0085 (11)	0.0037 (12)	0.0039 (10)
C20	0.0495 (15)	0.0209 (11)	0.0278 (11)	−0.0015 (11)	−0.0011 (11)	−0.0001 (9)
C21	0.0360 (12)	0.0211 (11)	0.0292 (11)	0.0013 (10)	0.0009 (10)	−0.0008 (9)
C22	0.0680 (19)	0.0239 (12)	0.0369 (13)	0.0025 (12)	−0.0068 (13)	0.0040 (10)

### Geometric parameters (Å, º)

**Table:**

O1—N2	1.482 (2)	C14—C15	1.550 (3)
O1—C9	1.424 (3)	C14—C18	1.531 (3)
N2—C3	1.493 (3)	C14—H141	0.997
N2—C7	1.489 (3)	C15—C16	1.524 (3)
C3—N4	1.472 (3)	C15—C17	1.527 (3)
C3—C14	1.552 (3)	C15—H151	1.001
C3—C21	1.531 (3)	C16—H162	0.983
N4—C5	1.448 (3)	C16—H163	0.977
N4—C6	1.349 (3)	C16—H161	0.986
C5—H51	0.969	C17—H171	0.991
C5—H53	0.956	C17—H173	0.957
C5—H52	0.974	C17—H172	0.970
C6—C7	1.509 (3)	C18—C19	1.526 (4)
C6—O13	1.226 (3)	C18—H182	0.992
C7—C8	1.523 (3)	C18—H181	0.988
C7—H71	0.959	C19—C20	1.533 (4)
C8—C9	1.536 (3)	C19—H192	0.999
C8—C12	1.513 (3)	C19—H191	1.001
C8—H81	0.957	C20—C21	1.518 (3)
C9—C10	1.521 (3)	C20—C22	1.529 (3)
C9—H91	0.980	C20—H201	0.990
C10—O11	1.433 (3)	C21—H211	0.996
C10—H102	0.989	C21—H212	0.966
C10—H101	0.983	C22—H222	0.972
O11—C12	1.423 (3)	C22—H221	0.995
C12—H122	0.985	C22—H223	1.009
C12—H121	0.981		
			
N2—O1—C9	103.68 (14)	C3—C14—C18	110.82 (19)
O1—N2—C3	106.18 (15)	C15—C14—C18	112.69 (19)
O1—N2—C7	100.99 (15)	C3—C14—H141	103.9
C3—N2—C7	106.11 (15)	C15—C14—H141	105.9
N2—C3—N4	103.92 (16)	C18—C14—H141	107.2
N2—C3—C14	109.41 (17)	C14—C15—C16	109.79 (19)
N4—C3—C14	111.43 (18)	C14—C15—C17	114.5 (2)
N2—C3—C21	113.10 (17)	C16—C15—C17	109.3 (2)
N4—C3—C21	110.18 (18)	C14—C15—H151	108.1
C14—C3—C21	108.77 (18)	C16—C15—H151	106.8
C3—N4—C5	123.70 (17)	C17—C15—H151	108.1
C3—N4—C6	113.29 (17)	C15—C16—H162	108.5
C5—N4—C6	122.47 (19)	C15—C16—H163	112.6
N4—C5—H51	109.8	H162—C16—H163	108.7
N4—C5—H53	110.8	C15—C16—H161	108.4
H51—C5—H53	106.1	H162—C16—H161	110.3
N4—C5—H52	110.4	H163—C16—H161	108.3
H51—C5—H52	109.2	C15—C17—H171	107.4
H53—C5—H52	110.4	C15—C17—H173	110.8
N4—C6—C7	107.39 (18)	H171—C17—H173	109.1
N4—C6—O13	126.4 (2)	C15—C17—H172	112.4
C7—C6—O13	126.2 (2)	H171—C17—H172	110.5
C6—C7—N2	105.48 (16)	H173—C17—H172	106.5
C6—C7—C8	116.15 (19)	C14—C18—C19	113.0 (2)
N2—C7—C8	106.89 (17)	C14—C18—H182	106.7
C6—C7—H71	108.3	C19—C18—H182	110.9
N2—C7—H71	108.7	C14—C18—H181	107.8
C8—C7—H71	111.0	C19—C18—H181	109.5
C7—C8—C9	101.88 (16)	H182—C18—H181	108.9
C7—C8—C12	114.2 (2)	C18—C19—C20	110.74 (19)
C9—C8—C12	102.57 (18)	C18—C19—H192	108.4
C7—C8—H81	109.3	C20—C19—H192	107.8
C9—C8—H81	114.4	C18—C19—H191	110.1
C12—C8—H81	113.9	C20—C19—H191	110.4
C8—C9—O1	105.88 (16)	H192—C19—H191	109.2
C8—C9—C10	104.18 (18)	C19—C20—C21	109.3 (2)
O1—C9—C10	114.75 (19)	C19—C20—C22	111.8 (2)
C8—C9—H91	111.5	C21—C20—C22	110.0 (2)
O1—C9—H91	109.5	C19—C20—H201	109.0
C10—C9—H91	110.8	C21—C20—H201	108.1
C9—C10—O11	106.84 (18)	C22—C20—H201	108.7
C9—C10—H102	110.7	C3—C21—C20	113.52 (19)
O11—C10—H102	110.6	C3—C21—H211	107.3
C9—C10—H101	111.5	C20—C21—H211	107.5
O11—C10—H101	108.3	C3—C21—H212	108.2
H102—C10—H101	108.9	C20—C21—H212	110.3
C10—O11—C12	105.74 (19)	H211—C21—H212	109.9
C8—C12—O11	104.57 (19)	C20—C22—H222	111.5
C8—C12—H122	111.7	C20—C22—H221	109.4
O11—C12—H122	112.4	H222—C22—H221	108.8
C8—C12—H121	111.6	C20—C22—H223	111.6
O11—C12—H121	109.7	H222—C22—H223	106.8
H122—C12—H121	107.0	H221—C22—H223	108.7
C3—C14—C15	115.54 (18)		
			
C9—O1—N2—C3	156.12 (16)	C21—C3—C14—C18	53.8 (2)
C9—O1—N2—C7	45.58 (18)	N2—C3—C21—C20	64.1 (2)
N2—O1—C9—C8	−40.8 (2)	N4—C3—C21—C20	179.91 (18)
N2—O1—C9—C10	73.5 (2)	C14—C3—C21—C20	−57.7 (2)
C12—O11—C10—C9	−30.4 (2)	O13—C6—C7—N2	−169.1 (2)
C10—O11—C12—C8	41.4 (2)	O13—C6—C7—C8	−51.0 (3)
O1—N2—C3—N4	−88.72 (18)	N4—C6—C7—N2	13.2 (2)
O1—N2—C3—C14	152.15 (15)	N4—C6—C7—C8	131.3 (2)
O1—N2—C3—C21	30.7 (2)	N2—C7—C8—C9	9.3 (2)
C7—N2—C3—N4	18.2 (2)	N2—C7—C8—C12	−100.5 (2)
C7—N2—C3—C14	−100.94 (18)	C6—C7—C8—C9	−108.1 (2)
C7—N2—C3—C21	137.64 (18)	C6—C7—C8—C12	142.1 (2)
O1—N2—C7—C6	91.23 (18)	C7—C8—C9—O1	19.2 (2)
O1—N2—C7—C8	−32.94 (19)	C7—C8—C9—C10	−102.2 (2)
C3—N2—C7—C6	−19.4 (2)	C12—C8—C9—O1	137.68 (18)
C3—N2—C7—C8	−143.56 (17)	C12—C8—C9—C10	16.3 (2)
C5—N4—C3—N2	177.7 (2)	C7—C8—C12—O11	74.2 (2)
C5—N4—C3—C14	−64.6 (3)	C9—C8—C12—O11	−35.1 (2)
C5—N4—C3—C21	56.2 (3)	O1—C9—C10—O11	−107.8 (2)
C6—N4—C3—N2	−10.6 (3)	C8—C9—C10—O11	7.6 (2)
C6—N4—C3—C14	107.2 (2)	C3—C14—C15—C16	146.3 (2)
C6—N4—C3—C21	−132.0 (2)	C3—C14—C15—C17	−90.4 (2)
C3—N4—C6—O13	−179.3 (2)	C18—C14—C15—C16	−84.9 (2)
C3—N4—C6—C7	−1.6 (3)	C18—C14—C15—C17	38.4 (3)
C5—N4—C6—O13	−7.4 (4)	C3—C14—C18—C19	−54.8 (3)
C5—N4—C6—C7	170.3 (2)	C15—C14—C18—C19	174.06 (19)
N2—C3—C14—C15	59.5 (2)	C14—C18—C19—C20	55.5 (3)
N2—C3—C14—C18	−70.2 (2)	C18—C19—C20—C21	−55.6 (3)
N4—C3—C14—C15	−54.8 (2)	C18—C19—C20—C22	−177.5 (2)
N4—C3—C14—C18	175.48 (17)	C19—C20—C21—C3	58.7 (2)
C21—C3—C14—C15	−176.47 (18)	C22—C20—C21—C3	−178.24 (19)

### Hydrogen-bond geometry (Å, º)

**Table:**

*D*—H···*A*	*D*—H	H···*A*	*D*···*A*	*D*—H···*A*
C5—H52···O13^i^	0.97	2.57	3.536 (3)	172

Symmetry code: (i) −*x*+1, *y*−1/2, −*z*+1/2.
